# Should Health Professionals Screen All Women for Domestic Violence?

**DOI:** 10.1371/journal.pmed.0010004

**Published:** 2004-10-19

**Authors:** Ann Taket, C. Nadine Wathen, Harriet MacMillan

## Abstract

Background to the debate: The US and Canadian task forces on preventive health recently declared that there is not enough evidence to recommend for or against routine universal screening of women for domestic violence. Yet some experts argue that routine enquiry is justified.

## Abstract 2

Experts from both sides of the Atlantic debate whether screening can be justified based on the available clinical evidence

## Ann Taket's Viewpoint: Routinely Asking about Domestic Violence Is Worthwhile

Domestic violence is a misunderstood topic. The context of a trusted health professional talking to a woman is one that provides an important opportunity for providing information to counter misconceptions.

I deliberately talk about this in terms of *asking* all women about domestic violence and not in terms of *screening* women for domestic violence. It is not appropriate or helpful to regard enquiry about being abused as a form of screening. Domestic violence is not a disease present in the body of the person who experiences it—rather it is a health-related risk factor.

As such, knowledge of abuse puts health professionals in a position to respond better to the needs of women affected by it. Professionals can respond by providing information on specialist services—usually provided outside the health service—that women may access if they wish. By giving information to affected women, health professionals can also help to reduce women's sense of isolation and stigmatisation. Asking about experience of domestic violence can be seen as a routine part of history taking, just as health professionals regularly and repeatedly ask patients about their smoking behaviour, alcohol use, weight, and exercise.

The prevalence of domestic violence among women is such that, even if it is not a personal issue for the woman concerned, it most likely will be for one or more of her relatives, friends, and neighbours [1]. Since many women experiencing abuse feel alone and ashamed, and their abusers often encourage them to believe that the abuse is their fault, presenting information to counter women's negative feelings is an important preventive strategy.

Most women experiencing domestic violence report that the specialised services that exist to respond to their needs were difficult to find out about [2]. The provision of simple information on the existence of specialised services and how to contact them is relevant to all women.

Studies have examined women's views on being asked about domestic violence. These studies have shown that once they have experienced being asked, they are usually in favour of being asked. This is true both for those who have experienced or are experiencing abuse, and those who have not [3]. It is only a small minority of women who object to being asked, or who find the question uncomfortable. Women who have experienced abuse particularly value being asked directly.[Fig pmed-0010004-g001]

**Figure pmed-0010004-g001:**
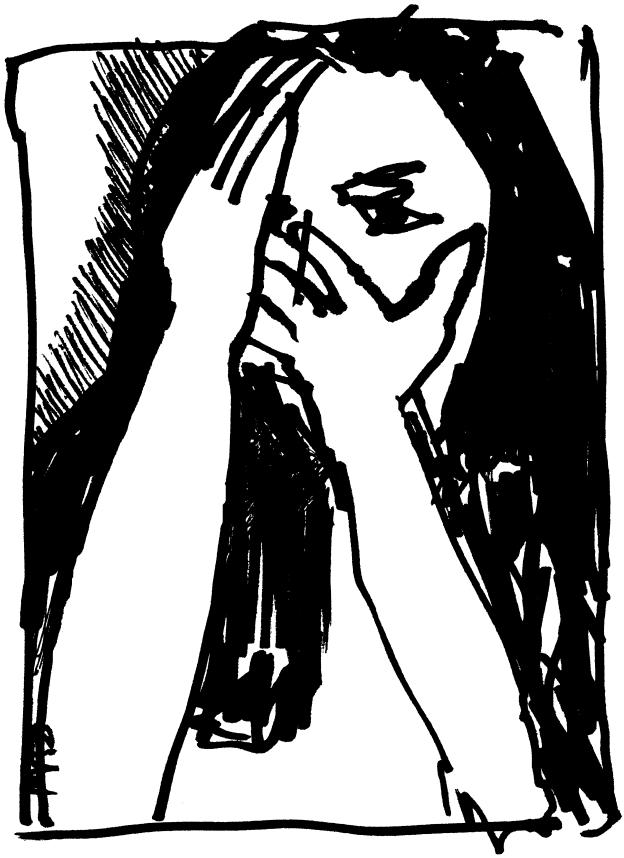
Women experiencing violence often feel alone and ashamed (Illustration: Margaret Shear, Public Library of Science)

Asking about abuse should be done in a flexible fashion—the particular questions used should respond to the circumstances of the consultation. For example, it is appropriate to ask women about domestic violence as part of a health check in a Well Woman Clinic, but it would be completely inappropriate in a consultation where another adult or a child was present. By being flexible, health professionals can integrate their questioning within a variety of different encounters. Integrating questions about abuse into routine encounters provides for the maintenance of confidentiality and safety. In order to do this, health professionals require training on raising the issue and knowledge about local advice and support services.

Committees on both sides of the Atlantic have rejected the notion of screening women for domestic violence, arguing that there is insufficient evidence of the effectiveness of interventions [4,5]. Part of the reason for this lack of evidence is that the systematic reviews on which these committees based their recommendations often excluded the most important types of evidence that do exist [3,6]. For example, these reviews excluded studies done outside the health service setting—they excluded those based in social services, or in the voluntary or community sector. Some excluded studies show the effectiveness of specialised service provision for women experiencing abuse.

In one example of an excluded study, researchers used a randomised design to evaluate an advocacy service for women experiencing domestic violence [7,8]. Women were interviewed six times over two years, and women in the intervention group reported a higher quality of life, decreased difficulty in obtaining community resources, and less violence over time than women in the control group. Other studies showing the value of specialised support services provided outside of the health system provide evidence of the potential benefits of asking about abuse [2].

Systematic reviews have also excluded, or devalued, evidence from qualitative studies. For example, a study of 200 women who had used domestic violence outreach services found that about half were living in situations of domestic violence when they first contacted the service. All of these women reported that the outreach services had helped them to leave the abusive relationship—a valued outcome for them [9].

Given the health impacts on women who experience domestic violence (not to mention their children) and the prevalence of the problem, routinely asking women about abuse should be seen as an important form of primary and secondary prevention for a wide range of health problems.

## Nadine Wathen and Harriet MacMillan's Viewpoint: The Decision to Screen Should Be Based on Evidence

Screening tools for domestic violence are abundant, and many are effective at identifying women experiencing abuse [3,10]. However, merely identifying a woman as abused has not been shown to actually improve her quality of life or reduce the violence she is experiencing [6,11]. Furthermore, with one exception [7], we do not know whether interventions for women exposed to violence are effective in reducing violence or improving other health-related outcomes. Interventions for abusive men have shown little effectiveness [11,12].

Given the morbidity and mortality associated with domestic violence, it is tempting to suggest that universal screening for abuse should be integrated into routine clinical care, such that all women, regardless of their reason for presenting to a clinical setting, should be “asked the question.” Some argue that this approach is justified by the need to increase awareness of domestic violence as a significant problem with serious health and social consequences, and to make abused women aware that they are not alone in their experience. These are important considerations.

Certainly all women who disclose that they have been exposed to violence should be provided with options regarding seeking help [13]. Good diagnostic assessment requires that clinicians be able to identify and respond to signs and symptoms of abuse, from patterns of physical injury to mental health concerns, including unexplained pain and depression. Not asking women about exposure to violence during certain diagnostic assessments (such as investigation of chronic pain) may lead to misdiagnosis and a path of inappropriate investigations or treatments that will miss the underlying problem [14]. It is also imperative that clinicians know about the hospital- or community-based services that exist and ensure that there is a system in place to provide appropriate referral [15].

However, what about women presenting *without* obvious signs and symptoms of domestic violence—such as a woman who comes to the clinic for assessment of an upper respiratory tract infection? Should such women be prompted to disclose whether they are being abused? The woman who is not being abused will answer to that effect, and the appointment can carry on. But for the woman who is experiencing violence, who has not volunteered this information, several factors must be considered. An important issue is whether she is ready—both psychologically and in terms of taking specific actions—to confront the issue. A number of excellent qualitative studies have examined the process that women undertake in acknowledging that they are “victims” of “abuse” and embarking on the often long and difficult journey to avoid, reduce, and ultimately stop the violence in their lives [16,17]. Given the enormousness of that task, the key question becomes the extent to which prompting disclosures of abuse through universal screening will actually help women in this process, and help them in a way that they find meaningful.

Any potential benefits of screening must then be weighed against its potential harms, including labelling women, prompting potentially premature disclosure, and triggering possible reprisal violence from the abuser if he discovers she has sought help. The last of these might be particularly exacerbated for the woman with the respiratory tract infection who was unprepared to disclose and did not take necessary precautions. Other potential harms include exposure to the ramifications of laws on mandatory child protection reporting, whereby health providers must report such disclosures to child protection authorities. This can lead to an investigation that potentially increases a woman's risk of exposure to violence, and in some cases of having her children placed in foster care. Research has shown that many of these potential harms are of concern to women when mandatory universal screening and/or reporting protocols are in place [18]. Finally, from a health system perspective, the opportunity cost of not having used this time with the woman to conduct screening or prevention activities for which there is proven benefit, such as counselling about Pap smears or mammograms, should not be discounted.[Fig pmed-0010004-g002]

**Figure pmed-0010004-g002:**
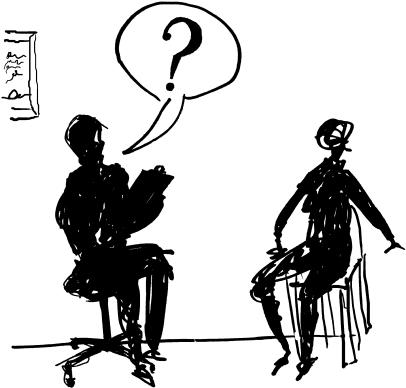
There are potential harms from “asking the question” (Illustration: Margaret Shear, Public Library of Science)

Given the lack of clear data on the benefits of screening and of the interventions to which women are referred, and the lack of data on potential harms, we and others have concluded the following [3,19,20]. Until these questions are answered, the most appropriate health care system approach is the more targeted case-finding or diagnostic method, which focuses health care resources on those in immediate need of care. Our hope is that studies currently underway (for example, those supported by the Ontario Women's Health Council and the US Centers for Disease Control) will provide information about the effectiveness of domestic violence screening. Let's base the decision about implementation of screening on evaluations of whether such screening does more good than harm in the lives of women.

## Taket's Response to Wathen and MacMillan's Viewpoint

I agree entirely with Nadine Wathen and Harriet MacMillan that practice should be based on evidence. There are further areas of agreement. We agree that there is a lack of knowledge on effective interventions for abusers and on harm occurring as a result of enquiry, and that targeted case finding is important.

The key difference that exists between my viewpoint and theirs is the conclusion about whether health professionals should aim to ask *all* women about domestic violence. Underlying this difference is the issue about how much evidence we need, and of what type. My position is that the evidence that already exists is sufficient to justify the promotion of routine enquiry, aiming to ask all women about their experience of abuse. There is evidence of actual benefits to women—and their children—from interventions provided by specialised services for domestic violence and from brief discussions with health professionals [21].

Aiming to ask all women has several advantages over targeted case finding [22]. It contributes to changing social attitudes to domestic abuse, it is less likely to make women experiencing abuse feel stigmatised, and it is less likely to compromise the safety of women experiencing abuse. Furthermore, health professionals report that their perceptions about which women are being abused, and which are not, are often wrong.

The twin issues of women's safety and harm minimisation are extremely important, for both routine enquiry and targeted case finding. These issues are important reasons why training and protocols for enquiry are necessary. Standard principles of confidentiality should be reinforced in training and protocols, which need to be tailored to relevant legal requirements, such as when child protection issues are involved. Training and protocols also need to emphasise that the role of routine enquiry is to facilitate, and not force, disclosure. It must remain the woman's choice as to if, when, and to whom, she discloses.

## Wathen and MacMillan's Response to Taket's Viewpoint

We agree with Ann Taket that domestic violence is not a disease, and that the paradigm of “screening for disease” is problematic in this context. At issue, however, is the question of whether domestic violence should be “talked about” with *all* women or only in situations where asking about it is part of a specific diagnostic assessment. As with screening for a disease, universal screening for domestic violence should not be implemented unless we are sure that interventions are available to help those identified via screening and that screening plus appropriate treatment will do more good than harm.

Professor Taket outlines the importance of integrating discussions about abuse in consultations to raise community awareness. Unfortunately, there is no evidence that this type of consciousness-raising occurs, or if it does, what benefit it might have. Given the lack of effectiveness of educational campaigns in general, it is difficult to be optimistic about this approach.

We disagree with her conclusion that existing systematic reviews have “excluded studies done outside the health service setting….” Our review included interventions such as the post-shelter advocacy counselling approach to which Professor Taket refers [11]. This intervention has been recommended by the Canadian Task Force on Preventive Health Care as one to which, where available, clinicians might refer women in these circumstances [19]. However, since shelters themselves have not been adequately evaluated, the value of linking screening to a post-shelter intervention is unclear.

Finally, we concur that qualitative studies are invaluable in understanding domestic violence. Such research has provided insight into the complex process that women undertake to address the violence in their lives. Until there is evidence that universal screening actually helps with this process, the focus should be on developing evidence-based approaches to assist women when they do disclose abuse and on training health professionals to respond appropriately to such disclosures.
